# Tcf7l2/Tcf4 Transcriptional Repressor Function Requires HDAC Activity in the Developing Vertebrate CNS

**DOI:** 10.1371/journal.pone.0163267

**Published:** 2016-09-26

**Authors:** Hui Wang, Michael P. Matise

**Affiliations:** 1 Department of Pharmacology, School of Pharmacy, Nantong University, Nantong, 226001, China; 2 Department of Neuroscience & Cell Biology, Rutgers-Robert Wood Johnson Medical School, 675 Hoes Lane, Piscataway, New Jersey, 08854, United States of America; Oxford Brookes University, UNITED KINGDOM

## Abstract

The generation of functionally distinct neuronal subtypes within the vertebrate central nervous system (CNS) requires the precise regulation of progenitor gene expression in specific neuronal territories during early embryogenesis. Accumulating evidence has implicated histone deacetylase (HDAC) proteins in cell specification, proliferation, and differentiation in diverse embryonic and adult tissues. However, although HDAC proteins have shown to be expressed in the developing vertebrate neural tube, their specific role in CNS neural progenitor fate specification remains unclear. Prior work from our lab showed that the Tcf7l2/Tcf4 transcription factor plays a key role in ventral progenitor lineage segregation by differential repression of two key specification factors, Nkx2.2 and Olig2. In this study, we found that administration of HDAC inhibitors (Valproic Acid (VPA), Trichostatin-A (TSA), or sodium butyrate) in chick embryos *in ovo* disrupted normal progenitor gene segregation in the developing neural tube, indicating that HDAC activity is required for this process. Further, using functional and pharmacological approaches in vivo, we found that HDAC activity is required for the differential repression of Nkx2.2 and Olig2 by Tcf7l2/Tcf4. Finally, using dominant-negative functional assays, we provide evidence that Tcf7l2/Tcf4 repression also requires Gro/TLE/Grg co-repressor factors. Together, our data support a model where the transcriptional repressor activity of Tcf7l2/Tcf4 involves functional interactions with both HDAC and Gro/TLE/Grg co-factors at specific target gene regulatory elements in the developing neural tube, and that this activity is required for the proper segregation of the Nkx2.2 (p3) and Olig2 (pMN) expressing cells from a common progenitor pool.

## Introduction

Cell fate specification in the ventral neural tube of the vertebrate CNS involves the translation of graded extracellular Sonic hedgehog (Shh) signaling into discrete progenitor territories that generate specific neuronal and glial subtypes [1–3]. Homeodomain (HD) containing transcription factor proteins, many of which are directly regulated by Shh signaling, play a crucial role in this process by refining domain boundaries through a process of mutual cross-repression [4]. One common theme underlying their mechanism of action is that they recruit Gro/TLE/Grg co-repressors [5]. Gro/TLE family proteins have been widely studied for their role as transcriptional regulators [6]. These proteins contain five identified domains that mediate tetramerization and binding to other proteins, and are thought to function as bridging factors that help assemble transcription-regulating repressor complexes at enhancers [6]. The specific mechanisms by which Gro/TLE/Grg proteins mediate transcriptional repression appear to vary depending on the tissue or cell type, but have been shown to include both direct effects on the core transcriptional machinery as well as epigenetic regulation of histone methylation and/or acetylation [7,8]. It has also been shown that Gro/TLE/Grg recruit Class I histone deacetylase (HDAC) proteins in drosophila embryos and vertebrate cell lines, which may repress cognate transcription factor target genes by inducing chromatin compaction [9]. However, it remains unclear whether a similar mechanism accounts for Gro/TLE/Grg repression in other cell or tissue types.

In our prior studies, we identified the Tcf/Lef family protein Tcf7l2/Tcf4 (hereafter referred to as Tcf4) as a non-HD repressor of ventral Shh target gene expression in the developing spinal cord [10,11]. Notably, we showed that Tcf4 repression of Nkx2.2 at the pMN/p3 boundary required Gro/TLE/Grg activity, confirming that the repressive activity of Tcf/Lef proteins function via recruitment of these factors in the cellular context of the developing spinal cord [8]. In the current study, we investigated further the mechanisms by which Tcf4 and Gro/TLE/Grg proteins regulate progenitor gene expression in the developing CNS. Our data shows that Tcf4 repression of gene expression in the ventral spinal cord requires HDAC activity and therefore supports a model whereby this factor functionally interacts with both Gro/TLE/Grg and HDAC proteins at specific Tcf/Lef target genes in the CNS.

## Materials & Methods

### In ovo electroporation and constructs

Fertilized specific pathogen-free (spf) White Leghorn chicken eggs (Charles River, Inc.) were incubated for 48 hours/H&H stage 12–13. Electroporation was carried out as previously described (Lei et al., 2004). Transfected embryos were returned to the incubator for about 26 hours and collected on E3/stage 18–19.

Grg4 deletion constructs were generated by PCR amplification from a full-length mouse Grg4 cDNA and encoded the following sequences: “Grg4-Q domain”, amino-acids 1–130; “Grg4-ΔQ domain” amino-acids 131–766. Both truncation constructs were cloned into the pCS2MT vector containing 5 myc epitope tags. Gli2A (ΔN-Gli2) and Tcf4R (ΔN-Tcf4) were described previously [10,12]. Full-length chick Nkx2.2 cDNA was cloned into pcDNA3 vector.

### Pharmacological inhibition of HDAC activity in vivo

To inhibit HDAC proteins in ovo, Valproic Acid (VPA, Sigma), Trichostatin-A (TSA, Sigma), or sodium butyrate (Sigma) diluted in L-15 media were applied to wild-type or transfected chick embryos at embryonic day (E) 2/ H&H stage 12 through a window made in the eggshell. Embryos were collected at stage 18–19 (26 hour post VPA application). Stock concentration of VPA was 1M used at 100mM (1:10), 10mM (1:100), and 1mM (1:1000). Stock concentration of TSA was 1mM used at 10μM (1:100), 1μM (1:1000), and 100nM (1:10000). Stock concentration of sodium butyrate was 100mM used at 10mM (1:10), 1mM (1:100), and 100μM (1:1000).

### Immunohistochemistry

Frozen tissue blocks were sectioned at 14 μm and mounted on Fisher permafrost slides. Immunohistochemistry using fluorescent labeled antibodies was performed as described [13]. Primary antibodies used were: mouse α-Nkx2.2 (DSHB 1:100), α -Foxa2 (DSHB, 1:100), and α-Myc (DSHB 1:200); rabbit α -Olig2 (1:6000; a gift from H. Takebayashi, Kyoto University, Kyoto, Japan), and α -Nkx2.2 (a gift from Tom Jessell, 1:4000); chicken α-GFP (Abcam 1:1000).

### Sequential chromatin immunoprecipitation (ChIP)

Sequential ChIP was performed with a Re-ChIP-IT Kit according to the manufacturer’s instructions (Active Motif). Antibodies used were: α-AcHistone3 (EZ-ChIP kit, Upstate), α-Tcf3/4 (6F12-3 ChIP grade, Abcam), α-HDAC1 and rabbit IgG control (ChIPAB+, Millipore). For Re-ChIP assays, ChIPs were first performed with α-HDAC1 antibodies, rabbit IgG (negative control group), or α-AcHistone3 (positive control group). The second immunoprecipitation was then performed with rabbit α-Tcf3/4 antibodies, rabbit IgG (negative control group), or α-AcHistone3 (positive control group). For one group of Re-ChIP assay, tissues used were one littermate of fresh Swiss Webster whole embryos collected at stage E10.5 (about 15 embryos 150–200mg) and optimal sheared chromatin weights 30–35ug. Tissues were fixed with freshly-made 10% paraformaldehyde solution for 15 minutes at room temperature. Chromatin was sheared by sonication into a size peaking about 500bp. PCR was performed using the following primers flanking the known (Nkx2.2) and (Olig2) Tcf consensus binding sequences: Nkx2.2, 5’-AGTATGTGACGTGGGTGACAATGG-3’ forward, 5’-GCCATGACAACTAGGGACAACCTT-3’ reverse; Olig2, 5’-GTTGTCTCTCTGGGTGGAAAGAGG-3’ forward and 5’-GGTGGGAAACGACAATGGTCCTTC-3’ reverse. Statistical analysis of sequential ChIP PCR data was performed by measuring the density of bands with ImageJ. Relative density was calculated using input bands as standards to normalize HDAC1 (primary IP) and Tcf3/4 (re-IP) readings.

## Results

### HDAC activity is required for ventral progenitor domain boundary formation and Tcf repressor patterning activity

Our prior studies provided evidence that Tcf4 repression of *Nkx2*.*2* in vivo involves a Gro/TLE/Grg dependent transcriptional repressor mechanism [12]. Gro/TLE/Grg co-repressors have been shown to recruit Class I histone deacetylase proteins (HDACs) that function to silence gene expression through modification of DNA/chromatin compaction [6,8,14,15]. These data raise the possibility that Tcf repression of Nkx2.2 may involve a similar mechanism involving HDAC.

**Fig 1:**
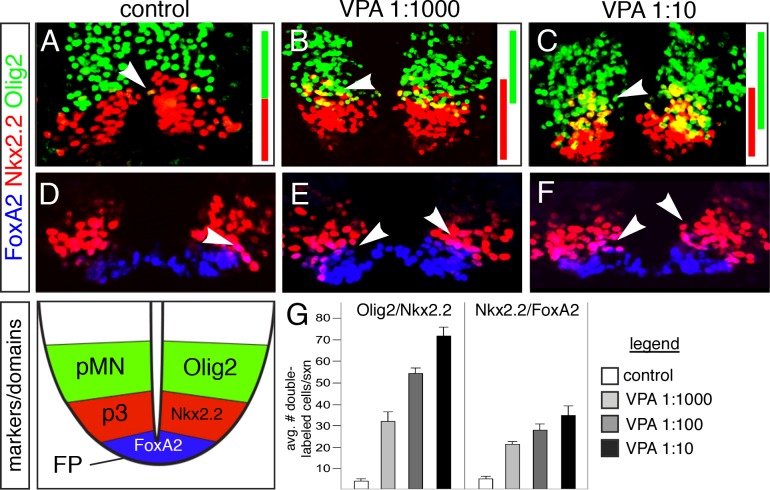
The histone deacetylase (HDAC) inhibitor VPA disrupts ventral progenitor boundaries. (A-F) Sections taken from E3 embryos treated with the indicated dilutions of VPA at E2 and stained for markers (Olig2, Nkx2.2, and FoxA2) that identify the three normally distinct ventral progenitor domains in the spinal cord. Inset schematic at bottom indicates the three progenitor domain boundaries analyzed and the corresponding markers used. pMN = motoneurons progenitor domain, p3 = V3 interneuron progenitor domain, FP = floor plate domain. Note that the number of double-labeled cells in both boundary regions increases significantly with increasing VPA concentrations (arrowheads) (A, D) controls; (B, E) VPA at 1mM; (C, F) VPA at 100mM). (G) Quantification of the results in A-F. *p<0.001.

To test this, we first assayed whether HDAC activity was necessary for ventral progenitor patterning by examining Nkx2.2 and Olig2 expression after blocking endogenous HDAC activity in the spinal cord with Valproic Acid (VPA) [16], since prior studies have shown that numerous Class I HDACs are expressed in the developing chicken spinal cord [17]. Consistent with this, we found that the normally sharp boundary between motoneuron and V3 subclass interneuron progenitor domains (termed the “pMN” and “p3”, respectively) was progressively disrupted at increasing VPA concentrations 26 hours after treatment at E2, with many cells co-expressing Olig2 and Nkx2.2 and mixing into each domain (Fig 1A–1C and 1G). Similar results were obtained using 2 additional HDAC inhibitors, Trichostatin A and Sodium Butyrate (S1 Fig). These results are consistent with the idea that endogenous Nkx2.2 cannot repress Olig2 in the p3 domain, which occurs via a Gro/TLE/Grg-dependent mechanism [5], under conditions of attenuated or blocked endogenous HDAC function. In support of this, repression of Olig2 in the pMN domain by electroporation (EP) of Nkx2.2 was also blocked by VPA (S2 Fig). Additionally, the number of cells co-expressing Nkx2.2 and FoxA2 (marking floorplate (FP) cells) increased with VPA dosage, indicating a similar HDAC-activity dependence of cross-repression at the p3/FP boundary (Fig 1D–1G). HDAC inhibition did not affect progenitor proliferation or neural differentiation rates, as judged by examining mitotic (pHH3) and pan-neural post-mitotic marker protein (NeuN) expression in VPA treated embryos (data not shown). These data indicate that normal HDAC activity is required for the HD-mediated cross repression that refines the pMN/p3/FP domain boundaries in the developing neural tube.

**Fig 2:**
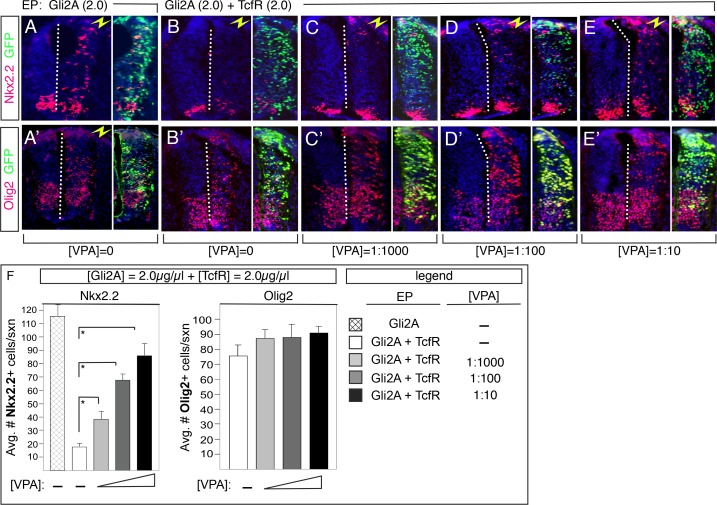
HDAC activity is required for Tcf repression of Gli2A-induced Nkx2.2 expression in vivo. (A-E) Sections through E3 chick embryos electroporated with Gli2A (at 2.0 μg/μl) and Gli2A+Tcf4R (at 2.0 μg/μl) in the presence of increasing concentrations of VPA. Right side was transfected in all cases. Note that the number of induced Nkx2.2+ cells increases with increasing VPA, indicating that Tcf4R antagonism becomes less effective at higher concentrations. (A’-E’) Olig2 expression in embryos transfected with Gli2A and Gli2A+TcfR in the presence of increasing concentrations of VPA. No effect is seen on Olig2 expression. (F) Quantification of data in A-E’. Inset at bottom right indicates corresponding bar chart shading. *p<0.001.

We next tested whether the ability of Tcf4 to block Gli2-mediated induction of ectopic Nkx2.2 (Lei et al., 2006) required HDAC activity. For this, we co-expressed an N-terminally truncated Gli2 cDNA (Gli2A) that encodes a constitutive transcriptional activator [10], and an N-terminally truncated Tcf4 cDNA (Tcf4R) encodes a constitutive transcriptional repressor, together in the presence of increasing concentrations of VPA, and assayed the number of induced ectopic Nkx2.2+ cells. As the concentration of VPA increased, so did the number of Nkx2.2+ cells (Fig 2A–2F). Similar results were obtained with Trichostatin-A and sodium butyrate (S1 Fig). Importantly, VPA had no effect on Gli2A induction of Nkx2.2 (S3 Fig), indicating that its affect on patterning cannot be explained by changes in Gli1 acetylation (which would be predicted to have a positive effect on its transcriptional activity based on the results from a prior study [18]). Finally, no effect on Olig2 expression was detected at any VPA concentration (Fig 2A’–2F), consistent with our prior data showing that Tcf4R does not effectively repress Olig2 induction in these assays (Lei et al., 2006), and work from other labs [19]. Together, these data support the idea that Tcf4 repression of ectopic Nkx2.2 involves HDAC in the spinal cord.

**Fig 3:**
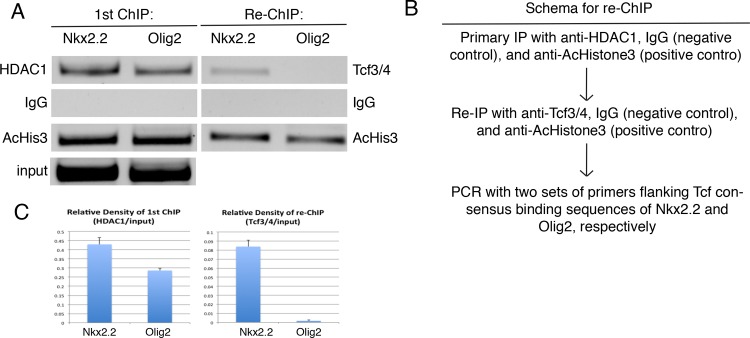
Tcf and HDAC1 proteins occupy Nkx2.2 but not Olig2 regulatory sequences. ChIP-reChIP experiments showing interaction of Tcf4 and HDAC1 with *Nkx2*.*2* but not *Olig2* regulatory regions. (A) Left column: Primary IP assays with HDAC1, IgG (negative control), or AcHistone3 (AcHis3) (positive control) antibodies from chromatin prepared from E10.5 mouse embryos. Right column: re-ChIP assays with Tcf3/4, IgG (negative control), or AcHistone3 (positive control) antibodies from primary IP elution. (B) Sequential chromatin immunoprecipitation assay schema. (C) Densitometry analysis of primary IP (left), and re-IP (right) results. The data are expressed as mean± SEM from three independent experiments.

These results raise the possibility that Tcf4 protein functionally interacts with HDAC1 at the *Nkx2*.*2*, but not *Olig2*, locus. To address this, we performed ChIP-reChIP experiments using the previously identified cis-regulatory sequences for these genes (*Nkx2*.*2*^*p3-CRM*^, *Olig2*^*pMN-CRM*^) [11,12,19] to first pull-down HDAC1 and then probe for the presence of Tcf4 protein (Fig 3). As predicted, both HDAC1 and Tcf4 could only be detected at the *Nkx2*.*2* ^*p3-CRM*^ (Fig 3). These findings are consistent with our data that indicate a selective functional interaction of Tcf4 with HDAC1 at the *Nkx2*.*2* but not *Olig2* locus.

### Groucho/TLE/Grg proteins link Tcf and HDAC activity

**Fig 4:**
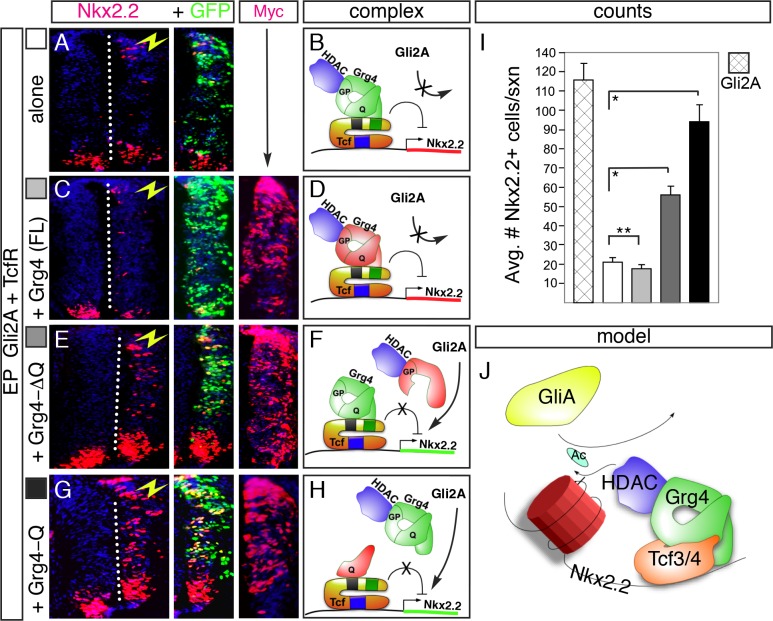
Functional interactions between Grg4 and HDAC/Tcf4 are required to repress Gli2A induction of Nkx2.2 in vivo. (A) Co-transfection of Tcf4R with Gli2A (both at (at 2.0 μg/μl) suppresses induction of Nkx2.2. (B) Predicted repressor complex for Tcf4R-mediated repression. (C, D) Co-transfection of a full-length Grg4 construct with Gli2A+TcfR also suppresses Nkx2.2 induction. (E-H) Co-transfection of Grg4 deletion constructs with Gli2A+TcfR. Both Grg4-ΔQ and Grg4-Q domain proteins prevent Tcf4R from blocking Gli2A-mediated induction of Nkx2.2 (seen in A). Antibody staining for the Myc-epitope tag was used to detect Grg4 constructs in all figures; GFP expression marks cells transfected with Tcf4R, while Gli activity was monitored by assaying Nkx2.2 expression. (I) Quantification of induced Nkx2.2 cells in each experiment, (J) Model for the regulation of Nkx2.2 by Tcf repressors involving HDAC activity and chromatin remodeling. *p<0.001, **p>0.05.

We next sought to establish whether Gro/TLE/Grg proteins act by bridging Tcf4 repressor and HDAC1 functions to regulate progenitor gene patterning in the ventral spinal cord. Gro/TLE/Grg factors are multi-domain proteins that do not bind directly to DNA but rather function to bring together transcription factors and other proteins in a repressor complex [8]. To do this, we performed transfection assays in chick embryos using full-length and truncated forms of the Grg4 protein, which was chosen on the basis of prior studies in mouse and chick that showed it is one of two Gro/TLE/Grg family genes expressed in ventral neural progenitors during neurogenesis in chicken and mouse embryos [5]. Co-transfection of a full-length Grg4 protein with Gli2A+Tcf4R did not block inhibition of Nkx2.2 (Fig 4C, 4D and 4I). We next co-transfected truncated forms of Grg4 that were missing key protein-protein interaction domains. We reasoned that mis-expression of a Grg4-ΔQ protein lacking the Tcf-binding and tetramerization “Q-domain” but retaining the “GP-domain” that binds to HDAC [6,8] along with Gli2A+Tcf4R should block the ability of Tcf4R to repress the induction of Nkx2.2 by dominantly interfering with the ability of endogenous Grg proteins to form a Tcf4-Grg-HDAC repressor complex at the endogenous *Nkx2*.*2* locus (see Fig 4F). Consistent with this, there was a significant increase in the number of Nkx2.2+ cells in triple-transfected embryos (Fig 4E and 4I) compared to controls transfected with Gli2A+Tcf4R alone (Fig 4A). Similarly, co-transfection of a Grg4 protein containing only the Q domain but not the GP domain (“Grg4-Q”) should also block complex formation by inhibiting the association of endogenous Grg proteins with Tcf4 (Fig 4H). As in the experiments above, a significant increase in the number of Nkx2.2+ cells were detected in triple-transfected embryos, compared to controls (Fig 4G and 4I). We also examined Olig2 expression as a control, and found no changes in any experiment (S4 Fig). Taken together, these results indicate that Tcf4 repressor activity requires functional association with both Grg and HDAC proteins, and suggest a model for how Tcf repressor activity selectively regulates the domain-restricted expression of Nkx2.2 via an HDAC-dependent mechanism, possibly involving chromatin modification (Fig 4J).

## Discussion

### A conserved mechanism for establishing progenitor domain boundaries in the CNS involving HDAC proteins

Deciphering the molecular genetic logic underlying patterned gene expression is fundamental to a better understanding of embryonic development and cell fate specification, and also has important implications for stem-cell directed therapies to treat human disease. In this study, using a combination of pharmacological, genetic, and functional assays, we show that the transcriptional repressor activity of Tcf7l2/Tcf4 in the developing CNS requires HDAC protein activity. Our data provides support for a functional interaction between Tcf4 and HDAC1 in repressing specific target gene expression at progenitor domain boundaries in the developing spinal cord. Our data also indicate that HDAC1 activity is required to mediate progenitor domain boundaries established by HD protein cross-repression. In addition to our functional data (shown in Fig 1 and S2 Fig), this idea is supported by results showing that HDAC1, but not Tcf4, was found to occupy the Olig2 regulatory sequences. We also provide evidence that Gro/TLE/Grg protein cofactors provide the link between the activities of these two factors, one a DNA-binding transcription factor and the second a protein modifying enzyme, to regulate gene expression in the CNS. Considered in the context of our prior genetic fate-mapping data in mice showing that cells contributing to these domains share a common lineage [11], these data indicate that a common mechanism underlies the transcriptional repressive activities of HD and other transcription factors that establish, sharpen and maintain FP/p3/pMN progenitor diversification and segregation the in the developing spinal cord

A number of prior studies in zebrafish have implicated HDACs in multiple critical signaling cascades during neural tube development, including Hedgehog (Hh), Wnt, Notch, and Fgf8 [20–24]. In the zebrafish hindbrain, HDAC1 has been shown to be required for proper neuronal and glial production (Cunliffe and Casaccia-Bonnefil, 2006). In these studies, reduction of HDAC1 function in the ventral neural tube resulted in deregulated expression of transcription factors nkx2.2a and olig2 and an impairment of oligodendrocyte specification, results which are consistent with our data. The current study extends prior work to reveal a molecular mechanism that mediates the regulation of specific HDAC-dependent ventral neural tube progenitor genes using chicken embryos, and suggests that a conserved mechanism underlies the role of HDAC in neuronal fate specification across phyla.

While our data do not rule out the possibility that HDAC activity may target non-histone proteins in the developing spinal cord, our results do not support the suggestion that modification of Gli protein activity via HDAC-mediated acetylation plays a significant role in regulating progenitor gene expression. Prior work showed that both Gli1 and Gli2 proteins were Class I HDAC targets in CNS tumor progenitor cells, and that de-acetylation promoted their transcriptional activities [18]. If a similar mechanism were in place in the developing neural tube, we would have expected that treatment with HDAC inhibitors (e.g., VPA) should result in an overall reduction in ventral Shh-Gli controlled gene expression, or an attenuation of the ability of Gli2 to induce ectopic target gene expression in transfection assays, neither of which was seen. Thus, our data support the interpretation that HDAC activity is primarily required for progenitor gene cross-repression during cell fate specification and patterning in the developing spinal cord.

## Supporting Information

S1 Fig The histone deacetylase (HDAC) inhibitors Trichostatin-A (TSA) and Sodium Butyrate (SB) disrupt ventral progenitor boundaries and Tcf repressor activity. (A-D) Sections taken from E3 embryos treated with the indicated dilutions of TSA (A, B) or SB (C, D) at E2 and stained for markers (Olig2 and Nkx2.2) that identify the pMN/p3 progenitor domains in the spinal cord. (E) Quantification of experiments shown in A-D show significant difference in the number of double-labeled cells compared to control for all dilutions (p<0.001). (F, G) Sections through E3 chick embryos electroporated with Gli2A (at 2.0 μg/μl) and Gli2A+Tcf4R (at 2.0 μg/μl) in the presence of TSA (1μM), or SB (1mM). (H) Quantification of data in F and G show significant difference in the number of Nkx2.2+ cells for both TSA and SB treatment compared to control condition (p<0.001).
(PDF)

S2 Fig Repression of Olig2 by Nkx2.2 requires HDAC activity. (A) Mis-expression of a full-length cNkx2.2 cDNA by electroporation (EP) in the pMN domain suppresses Olig2 protein expression in motoneurons progenitors. (B-C) The number of Olig2+ cells is greater with increasing amounts of VPA, indicating that inhibition of HDAC activity blocks Nkx2.2 repression of Olig2. (D) Quantification of results in A-C. *p<0.001.
(PDF)

S3 Fig Gli2 induction of Nkx2.2 does not require HDAC activity. Counts of Nkx2.2+ cells induced by transfection of Gli2A in the presence of increasing concentrations of VPA. A low level of Gli2A was chosen (0.1μg/μl) to provide the greatest sensitivity in assaying whether VPA can potentiate the activity of Gli2A. No effect was seen at any concentration tested. VPA concentrations are shown as dilutions and are the same for both sets of data in A and B. Bar chart shading key is shown to the right of the graph.
(PDF)

S4 Fig Control data for Tcf4/Grg4 co-transfections. (A) Olig2 expression in transfected with Gli2A+TcfR and Grg deletion constructs. No effect is seen on Olig2 expression in any experiment. (B) Quantification for experiments shown in A.
(PDF)
