# Supplementary figures and images for: Tcf7l2/Tcf4 Transcriptional Repressor Function Requires HDAC Activity in the Developing Vertebrate CNS
Source: PLoS One. 2016 Sep 26;11(9):e0163267. doi: 10.1371/journal.pone.0163267 (PMC5036887; doi:10.1371/journal.pone.0163267)

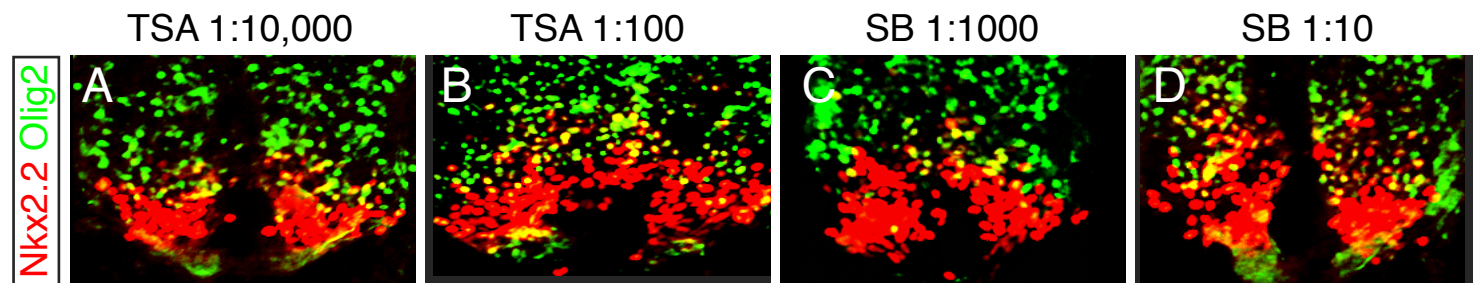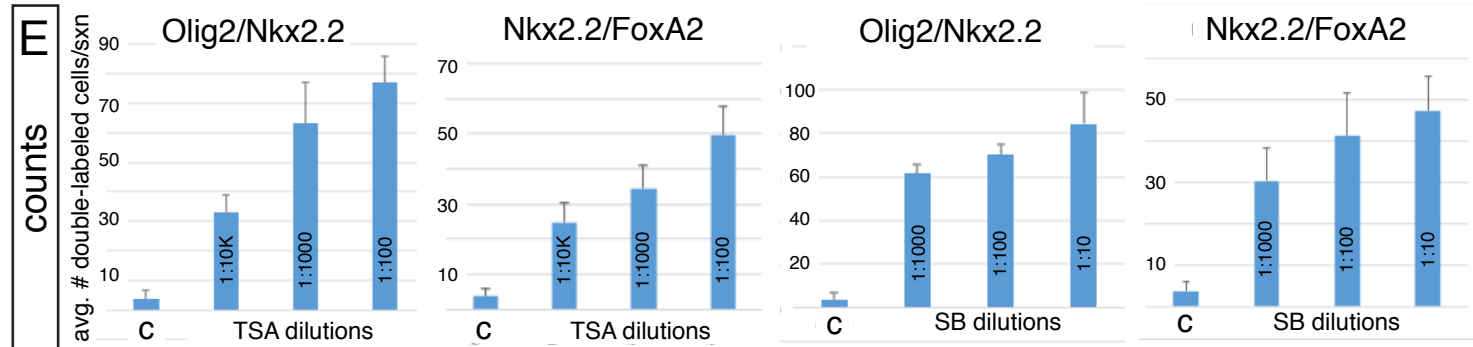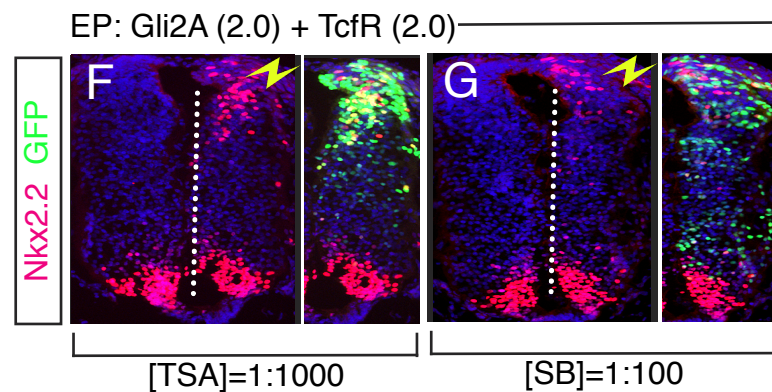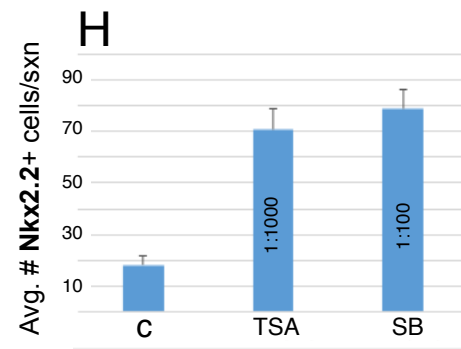

Supplement: S1 Fig — (A-D) Sections taken from E3 embryos treated with the indicated dilutions of TSA (A, B) or SB (C, D) at E2 and stained for markers (Olig2 and Nkx2.2) that identify the pMN/p3 progenitor domains in the spinal cord. (E) Quantification of experiments shown in A-D show significant difference in the number of double-labeled cells compared to control for all dilutions (p<0.001). (F, G) Sections through E3 chick embryos electroporated with Gli2A (at 2.0 μg/μl) and Gli2A+Tcf4R (at 2.0 μg/μl) in the presence of TSA (1μM), or SB (1mM). (H) Quantification of data in F and G show significant difference in the number of Nkx2.2+ cells for both TSA and SB treatment compared to control condition (p<0.001). (PDF) [file pone.0163267.s001.pdf]

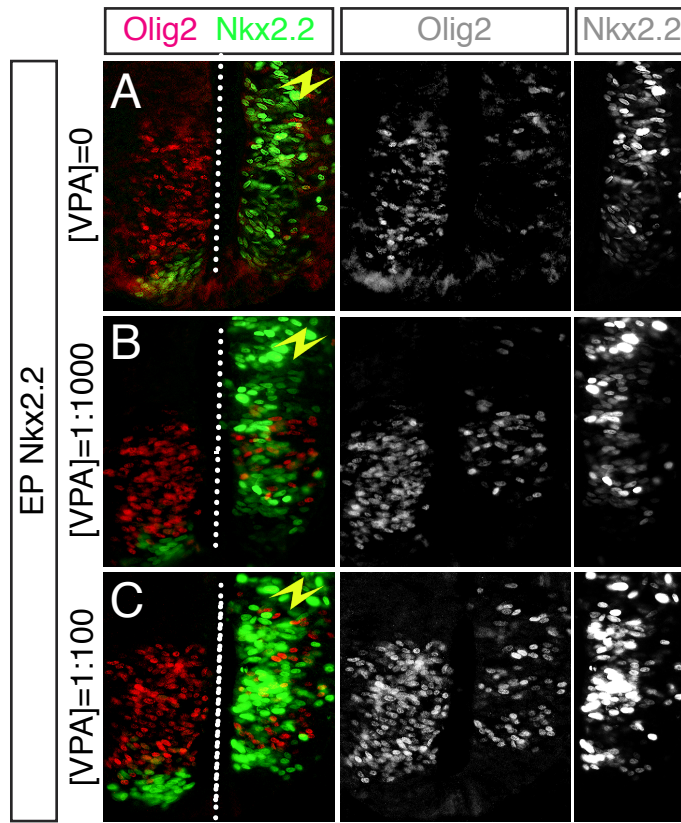

D

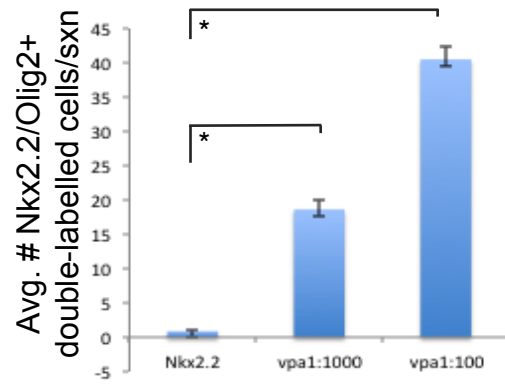

Supplement: S2 Fig — (A) Mis-expression of a full-length cNkx2.2 cDNA by electroporation (EP) in the pMN domain suppresses Olig2 protein expression in motoneurons progenitors. (B-C) The number of Olig2+ cells is greater with increasing amounts of VPA, indicating that inhibition of HDAC activity blocks Nkx2.2 repression of Olig2. (D) Quantification of results in A-C. *p<0.001. (PDF) [file pone.0163267.s002.pdf]

[Gli2A] = 0.1 $\mu$ g/ $\mu$ l

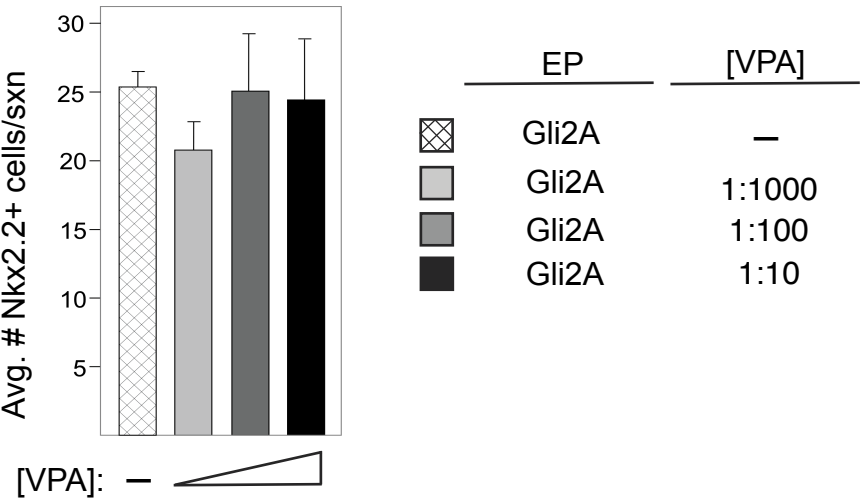

Supplement: S3 Fig — Counts of Nkx2.2+ cells induced by transfection of Gli2A in the presence of increasing concentrations of VPA. A low level of Gli2A was chosen (0.1μg/μl) to provide the greatest sensitivity in assaying whether VPA can potentiate the activity of Gli2A. No effect was seen at any concentration tested. VPA concentrations are shown as dilutions and are the same for both sets of data in A and B. Bar chart shading key is shown to the right of the graph. (PDF) [file pone.0163267.s003.pdf]

**A** EP: Gli2A (2.0) + TcfR (2.0)

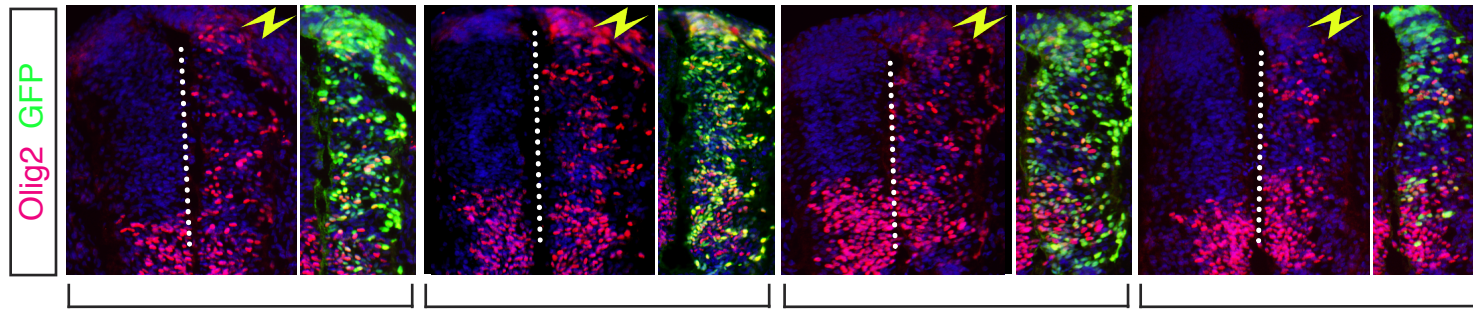

**B**

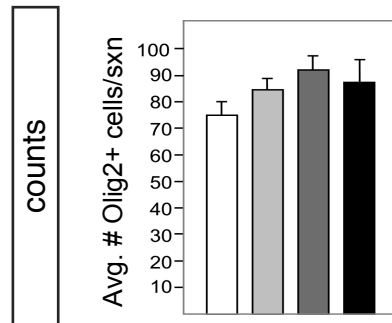

Supplement: S4 Fig — (A) Olig2 expression in transfected with Gli2A+TcfR and Grg deletion constructs. No effect is seen on Olig2 expression in any experiment. (B) Quantification for experiments shown in A. (PDF) [file pone.0163267.s004.pdf]
